# Intracranial halo pin penetration causing brain injury secondary to poor halo care technique: a case report and literature review

**DOI:** 10.1186/1757-1626-1-380

**Published:** 2008-12-09

**Authors:** Kishore Reddy Male, Abhijit Guha, Stuart James, Sashin Ahuja

**Affiliations:** 1 University Hospital of Wales, Cardiff, CF64 2HR, U.K.

## Abstract

This is a case report of intra cranial penetration by halo pins resulting in cerebritis and fits secondary to incorrect halo care by the patient and his family. Halo pin penetration into the skull with brain injury is itself a rare incident. Previously documented case reports were in patients with a previous cranioplasties and they were highlight the fact that halo not to be used in cranioplasty patients. Cranial penetration of the halo pins has generally been secondary to a fall/medical condition as epilepsy. This incident how ever highlights the fact the halo care itself along with proper techniques used for tightening the halo pins by the carer plays a crucial role in preventing complications such as this.

## Case presentation

This is a case report of a 39 year old young unemployed Caucasian restrained driver involved in a head on road traffic accident at 60 miles per hour sustaining an undisplaced type 2 fracture of the odontoid peg. A halo was applied using the torque wrench by the patient's bed side on the ward under aseptic precautions and discharged with an out patient follow up appointment. His partner kept tightening the screws with the torque wrench as and when required and 2 months down the line he presented with dizziness, fits and sickness. Computerized tomography showed that two screws penetrated his skull and findings consistent with temporal cerebritis. His halo was then changed into an aspen brace and he was treated conservatively for his cerebritis with intravenous antibiotics for 2 weeks followed by 6 weeks of oral antibiotics. He recovered fully without any complications and was discharged after 6 months of follow up. No follow up imaging was done for the cerebritis.

## Discussion

### Introduction

Using a Halo vest for temporary/definitive immobilisation and retention of unstable fractures of the upper cervical and unstable fractures at the cranio cervical junction is a common procedure being used[1]. Halo fixation decreases cervical movement by 30% to 96%[2]. Follow up of these patients with regards to appropriate halo care plays an equally crucial role in preventing any serious complications.

### Contraindications

Cranial fractures, intracranial injuries, soft tissue injuries of the skull and children < 3 years are all some of the major absolute contraindications for the procedure. Adiposity, chest injuries, paraplegia are the main relative contraindications[1].

### Technique

Application of a halo is done in two stages. The first one being application of a halo and then connecting it to a vest. Optimal size of the halo ring is normally 1.5" larger than the patients head circumference[1]. Threaded skull pins are used. The anterior ones are targeted into the shallow grove between the supraorbital ridges and frontal protuberences and these are screwed into the lamina externa of the cranial calotte with out perforating the lamina interna[1]. Diagonally opposite pins are tightened simultaneously to prevent any side-to-side rotation[1]. The torque used in application of the halo pins is very crucial to prevent the screws penetrating the inner table and that about 8 inch lb of torque is safe for the application of anterolateral pins and 18 inch lb for the posterolateral pins[3]. Pins should enter the skull perpendicular to the cortex. The posterolateral ones should pass about 1 cm above the helix of the ear[4]. The halo is then connected to the vest. The screws are usually tightened in the first 48 hours when they are prone to become loose using a torque wrench.

### Complications

The commonest complications seen are pin-loosening, pin site infections, pressure sores, pin-discomfort are the major one ones with high incidence[5]. Complications as nerve injury, dural penetration have been documented in literature but their incidence is quite low[5]. Note however is to be made that though their incidence is low these potentially serious complications can easily be prevented. Not many incidents of cranial penetration of a halo pin with associated brain injury have been previously reported[6].

## Conclusion

Intracranial penetration of the halo pins is a devastating complication which easily be prevented by appropriate halo care. If a patient is being discharged into the community with a halo on, then one should make sure that the person taking care of the halo in the community is trained appropriately to prevent any unforeseen complications.

## Consent

Written informed consent was obtained from the patient for publication of this case report. A copy of this written consent is available for review by the Editor – in – chief of this journal.

## Competing interests

The authors declare that they have no competing interests.

## Authors' contributions

KRM gathered data, analyzed data and played a key role in writing the case report. AG and SJ actively participated in literature review and writing the case report. SA supervised and guided us through the whole procedure whilst this was being written.
